# Stable magnesium peroxide at high pressure

**DOI:** 10.1038/srep13582

**Published:** 2015-09-01

**Authors:** Sergey S. Lobanov, Qiang Zhu, Nicholas Holtgrewe, Clemens Prescher, Vitali B. Prakapenka, Artem R. Oganov, Alexander F. Goncharov

**Affiliations:** 1 Geophysical Laboratory, Carnegie Institution of Washington, Washington, DC 20015, USA; 2 V.S. Sobolev Institute of Geology and Mineralogy SB RAS, Novosibirsk 630090, Russia; 3 Department of Geosciences, Department of Physics and Astronomy, Stony Brook University, Stony Brook, NY 11794, USA; 4 Howard University, 2400 Sixth Street NW, Washington, DC 20059, USA; 5 Center for Advanced Radiation Sources, University of Chicago, Chicago, IL 60632, USA; 6 Moscow Institute of Physics and Technology, 9 Institutskiy lane, Dolgoprudny city, Moscow Region, 141700, Russian Federation; 7 School of Materials Science, Northwestern Polytechnical University, Xi’an, 710072, China; 8 Key Laboratory of Materials Physics, Institute of Solid State Physics, CAS, Hefei, 230031, China; 9 University of Science and Technology of China, Hefei, 230026, China; 10 Skolkovo Institute of Science and Technology, Skolkovo Innovation Center, 5 Nobel St., Moscow 143026, Russia

## Abstract

Rocky planets are thought to comprise compounds of Mg and O as these are among the most abundant elements, but knowledge of their stable phases may be incomplete. MgO is known to be remarkably stable to very high pressure and chemically inert under reduced condition of the Earth’s lower mantle. However, in exoplanets oxygen may be a more abundant constituent. Here, using synchrotron x-ray diffraction in laser-heated diamond anvil cells, we show that MgO and oxygen react at pressures above 96 GPa and T = 2150 K with the formation of *I4/mcm* MgO_2_. Raman spectroscopy detects the presence of a peroxide ion (O_2_^2−^) in the synthesized material as well as in the recovered specimen. Likewise, energy-dispersive x-ray spectroscopy confirms that the recovered sample has higher oxygen content than pure MgO. Our finding suggests that MgO_2_ may be present together or instead of MgO in rocky mantles and rocky planetary cores under highly oxidized conditions.

Oxygen and magnesium are the first and second most abundant elements in the Earth’s mantle^1^; thus knowledge of stable phase relations in the Mg-O system as a function of thermodynamic parameters is necessary input information for reconstructing Earth-like planetary interiors. For example, ferropericlase (MgO with a relatively low Fe content) is the second most abundant mineral on Earth owing to its remarkable thermodynamic stability in the *Fm3m* crystal structure (up to 500 GPa and at least 5000 K for pure MgO)^2^,^3^. This is why ferropericlase has been assumed in gas giant cores^4^,^5^ as well as in extrasolar terrestrial mantles^6^,^7^. However, planet-harboring stars vary in chemical composition^8^, which likely affects the composition of planetary building blocks and exoplanet mineralogy^9^. Therefore, Earth-like mantle mineralogy should not be assumed for terrestrial exoplanets. Elevated oxygen contents have been observed in planet-host stars^10^, which may affect the stability of MgO and favor other solid phases in the Mg-O system^11^,^12^. For example, magnesium peroxide (MgO_2_) have been synthesized at near-ambient conditions and at high oxygen fugacities in the pyrite-type (*Pa3*) structure^11^. However, *Pa3* MgO_2_ is thermodynamically unstable and readily decomposes to MgO and O_2_ upon heating to 650 K at ambient pressure^11^. The intrinsic instability of MgO_2_ is attributed to the strong polarizing effect of the Mg^2+^ ion possessing high charge density in a relatively small ionic radius^13^. This is why the stability of Group II peroxides increases down the Group: beryllium peroxides are not known^13^, while Ca, Sr and Ba form increasingly more stable peroxides at ambient conditions^14^,^15^. Therefore, using empirical considerations on chemical pressure^16^,^17^ MgO_2_ may be expected to become stable under high pressure conditions. Indeed, *ab initio* simulations found that *I4/mcm* MgO_2_ becomes stable at P > 116 GPa (Ref. 12) and 0 K. Here, we report on the synthesis of *I4/mcm* MgO_2_ in a laser-heated diamond anvil cell (DAC). MgO_2_ may be an abundant mineral in highly oxidized terrestrial exoplanets. Our finding also suggests that the Mg-Fe-Si-O system likely has more unexpected chemistry at high pressure.

## Results

**Figure 1:**
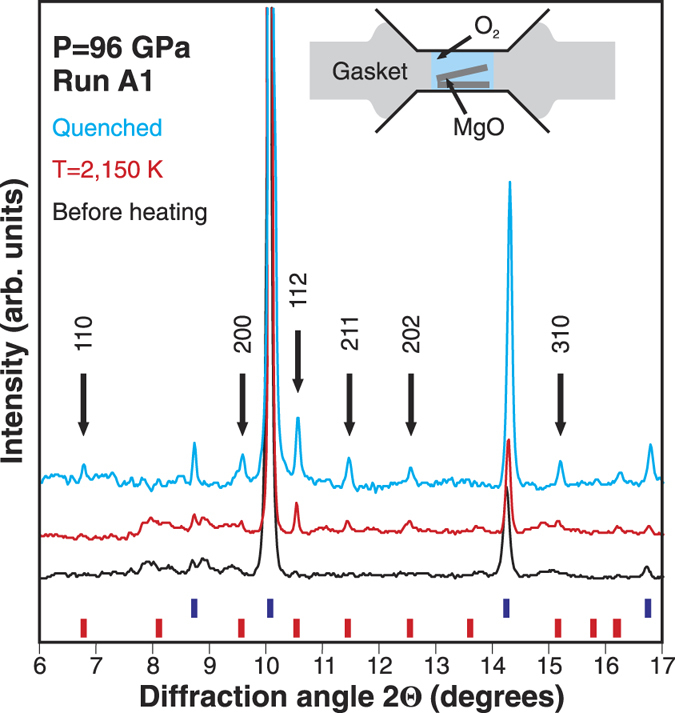
X-ray diffraction (XRD) pattern of the A1 sample before laser heating (black line), at high temperature (red line) and quenched to 300 K (blue line). Arrows mark new peaks that appear at high temperature. A thermal shift of the MgO peaks is seen at T = 2150 K indicating a uniform heating of the sample. Miller indices correspond to the indexed tetragonal unit cell. Expected positions of I4/mcm MgO_2_ Bragg reflections^12^ are shown by red ticks. Blue bars correspond to MgO. Oxygen peaks are not resolved. The wavelength is 0.3344 Å. The inset shows the experimental assemblage of type-A runs.

Two types of chemical precursors were loaded in DACs to study the MgO-O_2_ phase diagram in the 0–160 GPa pressure range (see Table 1 and Methods). In type-A experiments we put two 4 μm thick MgO disks in the sample cavity which was subsequently filled with liquefied oxygen (Fig. 1, inset). In type-B runs we used commercially available magnesium peroxide complex (24–28% *Pa3* MgO_2_, 42–46% MgO, ~30% Mg) mixed with submicron Au powder serving as a laser absorber. The mixture was loaded without pressure medium.

### X-ray diffraction

Figure 1 shows representative XRD patterns of the run A1 at 96 GPa before heating, at 2150 K, and after quenching. Oxygen peaks were weak and not resolved in the integrated pattern before laser-heating. Six new peaks appear upon heating and become clearly seen in the XRD pattern of the quenched sample. Indexing the new peaks reveals a tetragonal unit cell with *a* = 4.000(1) Å, *c* = 4.743(5) Å. The new peaks show a good match with the expected positions of the predicted *I4/mcm* MgO_2_ Bragg reflections^12^ (shown as red ticks in Fig. 1). Rietveld refinement of the new phase was not possible due to its spotty diffraction texture and because low intensity peaks could not be resolved (Supplementary Fig. S1).

In the experiments with type-B precursors MgO, ε-O_2_, and Au were the only phases observed in XRD patterns after it was heated to T > 2000 K in the pressure range of 5–70 GPa. Bragg peaks that can be assigned to *Pa3* MgO_2_ were completely absent in the reaction products suggesting that the precursor had decomposed to MgO and O_2_. Indeed, the presence of pure oxygen in the quenched sample was confirmed with Raman spectroscopy. Noteworthy, we did not observe elemental Mg (neither *hcp* at P < 50 GPa nor *bcc* at P > 50 GPa) in the reaction products. Magnesium likely reacts with oxygen as the latter gets liberated upon *Pa3* MgO_2_ decomposition at high temperature.

**Figure 2:**
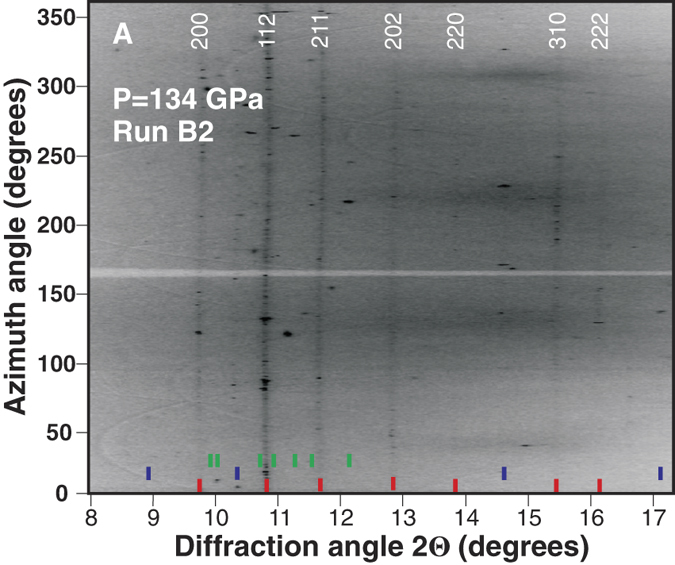
XRD image of I4/mcm MgO_2_ powder synthesized at 134 GPa (seen as dark grey vertical lines) in rectangular coordinates (cake). Red and violet ticks correspond to the positions of I4/mcm MgO_2_ and MgO, respectively. Green ticks represent some reflections of ζ-O_2_ (high angle Bragg reflections are not shown). White labels are Miller indices of the indexed tetragonal phase. Part of this XRD pattern (2θ = 9–13.5) was used to Rietveld refine the predicted structure of I4/mcm MgO_2_. The x-ray wavelength is 0.3344 Å.

**Figure 3:**
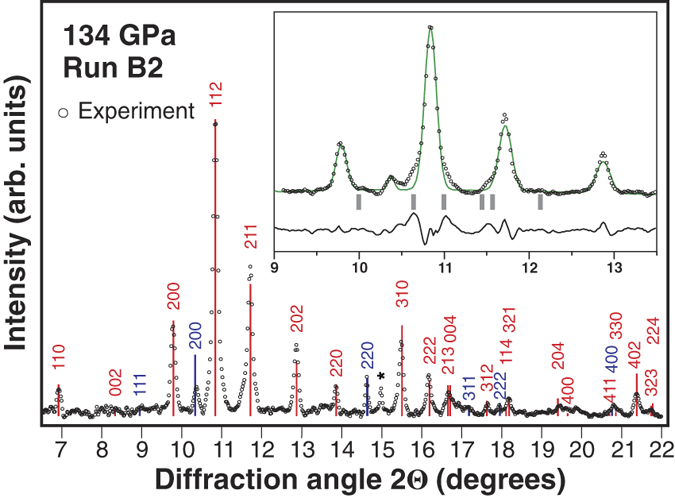
XRD of the type-B precursor laser-heated to T > 2000 K at 134 GPa. Red bars represent positions and intensities of Bragg reflections of the Rietveld refined I4/mcm MgO_2_. Dark blues bars correspond to MgO. The peak marked with an asterisk belongs to oxygen. **Inset:** Rietveld refinement of the MgO_2_ crystal structure. Grey bars approximate positions of the strongest ζ-O_2_ peaks. Green curve represents the calculated intensities of the refined structure (I_calc_). Black line is the intensity difference curve (I_obs_ − I_calc_). Calculated residuals after background subtraction are R_exp_ = 0.138, R_wp_ = 0.265. The x-ray wavelength is 0.3344 Å.

Laser heating of the B2 sample to T > 2000 K at P = 134 GPa provided more information on the high pressure chemistry of the Mg-O system. We were very curious to note that new peaks form a powder-type texture in XRD images (Fig. 2), indicating the presence of a large number of randomly oriented crystallites. Surprisingly, the spotty texture is now built by MgO and ζ-O_2_. Indexing the most clearly resolved new peaks again yields a tetragonal unit cell with *a* = 3.925 (1) Å, *c* = 4.613 (6) Å. Moreover, the obtained Miller indices reproduce that of the tetragonal phase synthesized in the A1 run (Fig. 1) suggesting that the exact same phase has been produced in the A1 and B2 runs. Given the large yields of the new phase as well as the polycrystalline sample texture, Rietveld method can be applied to test and refine the theoretically predicted *I4/mcm* MgO_2_. According to the prediction by Zhu *et al.* (Ref. 12), magnesium occupies a 4a Wyckoff position (*0, 0, 0.25*) and oxygen is located in 8 h (*x, x* + *0.5, 0*), which leaves only the *x* fractional coordinate of oxygen to refine. The refined *x* = 0.1285(13), and the predicted *x* = 0.126 agrees to within 2σ; thus the refined structural model may be considered identical to the predicted one. Figure 3 compares the experimental XRD pattern with the synthetic XRD of the Rietveld-refined *I4/mcm* MgO_2_.

**Figure 4:**
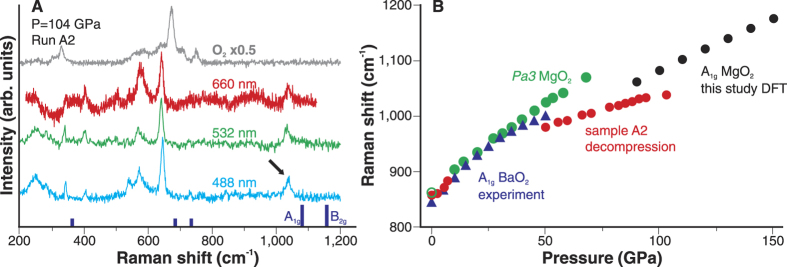
(**A**) Raman spectra of MgO + O_2_ reaction products collected with 488, 532, and 660 nm excitations. Oxygen Raman spectra collected outside of the laser-heated region is shown for comparison. Dark blue vertical ticks correspond to the computed Raman modes of I4/mcm at 100 GPa (A_1g_ and B_2g_ modes may have observable intensities, according to our DFT computations). The 1037 cm^−1^ peak that can be assigned to the A_1g_ mode in I4/mcm MgO_2_ is marked by an arrow. (**B**) Pressure dependencies of O-O symmetric stretching vibration (A_1g_). Red circles represent positions of the high-frequency mode observed in the A2 sample. Green circles correspond to the positions of the A_1g_ band in Pa3 MgO_2_ measured in this study and in Ref. 19 (green open circle at 1 atm.). Blue triangles are positions of the A_1g_ mode in BaO_2_. Black circles are computed frequencies of the A_1g_ mode in I4/mcm MgO_2_.

Raman spectroscopy was applied to characterize *I4/mcm* MgO_2_, albeit the increased fluorescent background of diamond anvils typical at pressures exceeding 100 GPa. On top of this, oxygen becomes metallic at pressure above 96 GPa^18^ and may screen reaction products from the probe laser radiation. First, we used density-functional perturbation theory (DFT) to compute spectral position and intensities of *I4/mcm* MgO_2_ Raman bands in the 90–150 GPa pressure range. Group theory for the *I4/mcm* MgO_2_ allows 5 Raman active vibrations (2E_g_ + B_1g_ + A_1g_ + B_2g_). Our DFT calculations suggest that B_2g_ and A_1g_ modes should have observable intensities with A_1g_ being the most intense as it may also be anticipated from earlier Raman studies of solid peroxides^19^. Figure 4A shows Raman spectra of A2 at 104 GPa collected from an area containing *I4/mcm* MgO_2_ as established by XRD. The O_2_ vibron was also observed in Raman spectra collected from the laser-heated spot. Since both ε- and ζ-O_2_ have a rich Raman spectrum^20^ at frequencies lower than 900 cm^−1^ it is difficult to use this spectral region for a reliable identification of the *I4/mcm* MgO_2_. Luckily, the position of A_1g_ band is predicted in the 1060–1175 cm^−1^ spectral range at 90–150 GPa according to our DFT calculations. Based on this comparison, the high-frequency mode at 1037 cm^−1^ may be assigned to the O-O stretching vibration in the peroxide ion. Raman shift of the high-frequency band is in agreement with the positions of A_1g_ band in H_2_O_2_ (Ref. 21) and BaO_2_ (Ref. 22) confirming the assignment.

Raman spectra of *I4/mcm* MgO_2_ were followed on A2 decompression run. In Fig. 4B the pressure-frequency dependence of the A_1g_ band of *I4/mcm* MgO_2_ is compared with that in BaO_2_ (Ref. 22) and *Pa3* MgO_2_ (this study and Ref. 19). We could only trace the high-frequency band down to 50 GPa, and then at 0–10 GPa because of the overlap with the overtone of oxygen L2 peak (2υ_L2_)^20^. Expectedly, the pressure dependence of the frequency O-O symmetric stretching in *Pa3* MgO_2_ is similar to that in *I4/mmm* BaO_2_. The DFT-computed frequencies of the A_1g_ in *I4/mcm* MgO_2_ also have a similar slope in the 90–150 GPa pressure range. However, the measured pressure dependence of the high-frequency band in the synthesized sample is less steep. Interestingly, at 1 bar the position of high-frequency band (857 cm^−1^) is almost identical to the position of A_1g_ mode in *Pa3* MgO_2_ (864 cm^−1^) (Ref. 19) suggesting that the recovered product is likely *Pa3* MgO_2_. Overall, our data provide spectroscopic evidence for the peroxide ion in the synthesized material and that the material containing peroxide ion is preserved to ambient conditions.

### Energy-dispersive x-ray spectroscopy

**Figure 5:**
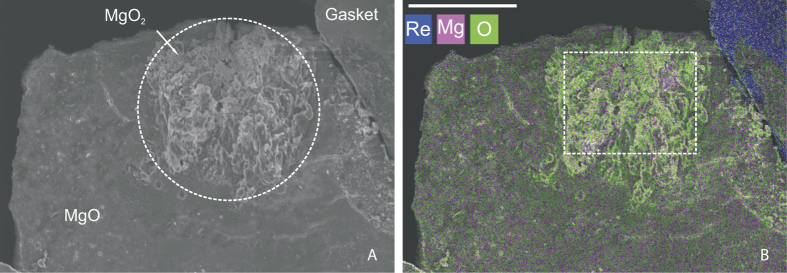
Electron microscope images of the extracted sample (run A2). (**A**) SEM micrograph. Laser-heated area is shown with a dashed circle. (**B**) Energy-dispersive x-ray spectroscopy image. Color intensity is proportional to the element abundance. The laser-heated area (white dashed line) has higher oxygen content. The white scale bar corresponds to 15 μm.

Mapping the extracted sample with an energy-dispersive x-ray spectroscopy (EDS) revealed that the laser-heated area has higher oxygen content (36 ± 2 at% Mg, 64 ± 3 at% O) than the area that was not subjected to high temperatures (Fig. 5). Detailed chemical characterization, however, was not possible because unreacted MgO is mixed with the oxygen-rich phase in the laser-heated area. Nevertheless, EDS analysis provides independent evidence for MgO_2_ in the recovered sample.

## Discussion

Bragg peaks of MgO_2_ were sharp in quenched samples right after the synthesis which allowed for a reliable volume determination with small σ values (Supplementary Table S1). On decompression, however, XRD peaks become broad probably due to the phase instability and volume measurements were less certain. Decompressed samples were mapped with the x-ray beam in order to find the best quality XRD, but only relative variations in Bragg peaks intensities were revealed. The new phase was still observed in XRD of the sample B2 decompressed down to 75 GPa. P-V data obtained on the sample B2 decompression is marked with an asterisk in the Supplementary Table S1. At P < 75 GPa the XRD peaks become too broad and start overlapping with peaks from other materials precluding identification of the MgO_2_ phase. Therefore, it remains unclear what physicochemical transformations occurred in the synthesized phase at P < 75 GPa. However, at 1 bar the laser-heated area of the recovered sample (A2) (Supplementary Fig. S2) shows a Raman signature of a peroxide ion with the Raman shift identical to that in *Pa3* MgO_2_.

**Figure 6:**
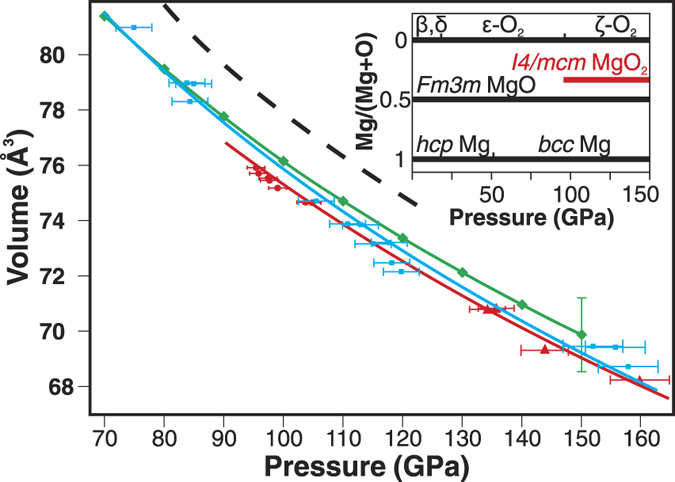
The 300 K third-order Birch-Murnaghan EOS of I4/mcm MgO_2_. Red line is EOS fit to the experimental data from runs A1, A2 (red circles), and B2 (red triangles) collected upon compression. Blue line is EOS fit to the experimental data collected on decompression (B2* in the Supplementary Table S1). The pressure error bar is based on the reported uncertainty of the MgO EOS (A-runs) and the maximum pressure difference between MgO and Au pressure gauges (B-runs). Green diamonds and green line are the DFT EOS of I4/mcm phase. Black dashed line is the sum of the unit cell volumes of MgO and O_2_ (taken with proper coefficients as dictated by the synthesis reaction and the number of formula units in the MgO and O_2_ unit cells). Inset: Experimental pressure-composition phase diagram of the Mg-O system as determined in this work. Stable phases are shown with thick solid lines.

Figure 6 shows a fit of the *I4/mcm* MgO_2_ P-V data collected upon compression (red line) and decompression (blue line) to the room temperature third-order Birch-Murnaghan equation of state (EOS). Sample annealing was not performed upon decompression which resulted in less precise P-V information. We also computed the *I4/mcm* MgO_2_ volume in the 70–150 GPa pressure range (Supplementary Table S2). The EOS parameters are reported in the Supplementary Table S3. The theoretically computed volumes are systematically 1.1% larger than the experimental ones in the 100–150 GPa pressure range, which is within the computational uncertainty.

*I4/mcm* MgO_2_ can be synthesized in the mixture of MgO with O_2_ at 96 GPa indicating a thermodynamic stability of MgO_2_ at this pressure, which is close to the theoretically predicted pressure of 116 GPa (Ref. 12), especially if one keeps in mind that the theoretical prediction was done at zero temperature. We therefore conclude that *I4/mcm* MgO_2_ is a thermodynamically stable phase in the high pressure phase diagram of the Mg-O system (Fig. 6, inset). The thermodynamic stability of *I4/mcm* MgO_2_ at P > 96 GPa is not surprising as heavier Group II elements, strontium and barium, form stable peroxides with CaC_2_-type (*I4/mmm*) crystal structure at ambient pressure with the O-O bond parallel to the *c* axis and 2 MO_2_ (M = Sr, Ba) formula units in the unit cell^14^. The O-O chemical bond length in MgO_2_ is 1.454 (1) Å at 96 GPa, which is comparable to that of SrO_2_ (1.483 Å) and BaO_2_ (1.493 Å) at standard conditions^14^. *I4/mcm* MgO_2_, however, has 4 formula units in the unit cell and the O-O bond is parallel to the *ab* plane diagonal (Supplementary Fig. S3A,B).

Taking into account that *Fm3m* MgO has 4 formula units and *C2/m* oxygen (ε−, ζ−) has 8 O_2_ molecules in the unit cell we calculated the volume of MgO + 1/2 O_2_ as a function of pressure using the reported MgO and O_2_ EOS^18^,^23^ (Fig. 6, dashed curve). It is apparent that *I4/mcm* MgO_2_ is denser than the reactants in the studied pressure range. Interestingly, the reaction of MgO with O_2_ at P > 96 GPa promotes an 8-fold coordination of Mg^2+^ at much lower pressures than expected for *Fm3m* to *Pm3m* (NaCl-type to CsCl-type) transition in pure MgO (∼500 GPa)^2^,^3^,^12^. In the *I4/mcm* phase of MgO_2_, there is a covalently bonded peroxo-group O_2_^2−^, ionically bonded with Mg^2+^ ions. The arrangement of Mg^2+^ and O_2_^2−^ ions is topologically identical to the CsCl structure type (Supplementary Fig. S3B,C).

*In situ* XRD at T = 2150 K (Fig. 1) demonstrates that MgO_2_ is stable at high temperature. Thus, MgO_2_ may be present together or instead of MgO in highly oxidized planetary interiors. Overall, the case of *I4/mcm* MgO_2_ shows that even the most inert planetary-forming minerals may be prone to chemical transformations.

## Methods

### Materials and samples

Diamond anvils with culets of 200, 300/100, and 300/80 μm were used to access the 100–160 GPa pressure range. Rhenium foils (200 μm thick) were indented to a thickness of 30–40 μm and then laser-drilled to create holes (30–100 μm in diameter) serving as sample chambers. Two types of chemical precursors were loaded in DAC to study the MgO-O_2_ phase diagram in the 0–160 GPa pressure range (see Table 1 and Fig. 1, inset). Magnesium oxide (99.85%) available from Alfa-Aesar was used for the type-A experiments. Before sample loadings magnesia was annealed at 1293 K for 12 hours to get rid of any adsorbed water. Two MgO disks were made by compressing the magnesia powder to a thickness of 4–5 μm and were stacked in the gasket hole. The remaining volume of the sample chamber was filled with liquefied zero-grade oxygen (99.8%, Matheson Gas Products) at approximately 77 K. In type-B experiments we used magnesium peroxide complex available from Sigma-Aldrich (24–28% *Pa3* MgO_2_, 42–46% MgO, ~30% Mg). The magnesium peroxide complex was mixed with submicron gold powder and loaded in the sample chambers with no pressure medium.

### Synthesis and characterization

All XRD experiments were performed at the undulator beamline at 13ID-D GeoSoilEnviroCARS, APS, using the online double-sided laser-heating system^24^. Oxygen exhibits strong near-infrared absorption at P > 10 GPa^25^,^26^,^27^ which allowed coupling the 1064 nm laser-heating radiation directly to oxygen in type-A experiments. Moreover, at P > 96 GPa oxygen turns metallic^18^,^20^,^28^ boosting the laser-heating efficiency. Finite element calculations reveal that diamond-sample interface remains at near-ambient temperatures almost independent of the sample and pressure medium, owing to diamond’s remarkable thermal conductivity^29^,^30^. Accordingly, no sign of etching was found on diamond anvils under an optical microscope after the experiments. In type-B runs, laser-heating radiation was coupled to the gold powder.

Synchrotron XRD was collected *in situ* at high temperature and high pressure in the diamond anvil cells to determine the onset of chemical and physical transformations with the x-ray beam (37.077 keV) focused to 4 μm spot size. Temperature was measured spectroradiometrically (Supplementary Fig. S4) simultaneously with XRD and calculated using the T-Rax software (C. Prescher). Temperature uncertainty of 150 K was assumed, typical of laser-heating DAC experiments^24^,^31^.

Mapping quenched samples with a step size of 5 (A runs) or 2 (B runs) μm to find areas with less O_2_ or Au, but with MgO was necessary for a careful indexing of the new phase XRD and to minimize the effect of pressure gradients across the sample chamber. MgO was present in both type-A and type-B experiments allowing consistent P-V measurements across the A- and B-runs. Tange *et al.*^32^ MgO pressure scale was preferred as it is based on several pressure-scale-free MgO thermodynamic data sets and allowed for minimal discrepancies with Au EOS^33^ in type-B experiments at 150–160 GPa. The maximum pressure differences observed between the MgO^32^ and Au^33^ EOS were on the order of 3–6 GPa (at 150 GPa), which was taken into account upon the *I4/mcm* MgO_2_ EOS fitting.

2D XRD patterns were integrated using the DIOPTAS software^34^. Manual background subtraction was done in Fityk (Ref. 35). Preliminary Bragg peaks indexing was performed with Dicvol06 (Ref. 36). GSAS/EXPGUI (Ref. 37, 38) was used for Rietveld refinement in accordance with the guidelines provided in Ref. 39, 40. Oxygen spotty reflections overlapping with the continuous lines produced by the new phase were masked. Also, we did not use the region of 2θ >13° where the background scattering is not uniformly distributed in the azimuth range of 0 to 360°. Scaling factors and unit cell parameters were refined first. Subsequently, peak profiles were fit with the pseudo-Voigt function and, at last, we refined the oxygen fractional coordinate (*x* in the 8 h position) of *I4/mcm* MgO_2_. Crystal structures were visualized with the use of VESTA 3 (Ref. 41). The 300 K third-order Birch-Murnaghan EOS was obtained using a (sigma)volume-weighted fitting procedure was performed as implemented in the EoSFit7GUI^42^.

Raman characterization of the quenched samples was performed in the Geophysical Laboratory. Solid-state lasers with 488, 532, and 660 nm lines focused to 3–4 μm were used as excitation sources. Backscattered Raman radiation was analyzed by a single-stage grating spectrograph equipped with a CCD array detector. The spectral resolution was 4 cm^−1^.

Our XRD and Raman data does not allow ruling out the formation of rhenium oxides at the gasket edge. However, the possible synthesis of rhenium oxides did not affect the careful characterization of *I4/mcm* MgO_2_. The tightly-focused x-ray beam allowed us analyzing reaction products within the laser-heated region and without sampling of the near-gasket regions. Likewise, Raman spectra assigned to the *I4/mcm* MgO_2_ were collected in a sample area shielded from Re by the oxygen rim (Supplementary Figure S2). At the same time, Raman data collected from the oxygen rim near the gasket had revealed only spectroscopic signatures of oxygen itself (Fig. 4A).

Energy-dispersive x-ray spectroscopy (EDS) analysis was performed on a dual beam focused ion beam/scanning electron microscope (FIB/SEM Zeiss Auriga 40) equipped with an Oxford X-Max 80 mm^2^ large-area silicon drift detector at the accelerating voltage of 5 kV in the Geophysical Laboratory. The analyzed sample was coated with Ir (~5 nm) to prevent specimen charging. Pyrope and the ENEL20 glass were used as standards for oxygen and magnesium, respectively.

Density functional theory (DFT) within the Perdew-Burke-Ernzerhof (PBE) generalized gradient approximation (GGA)^43^ as implemented in the VASP code^44^, was used for structural and vibrational analysis. For the structural relaxation, we used the all-electron projector-augmented wave (PAW) method^45^ and the plane wave basis set with the 600 eV kinetic energy cutoff; the Brillouin zone was sampled by Г-centered meshes with the resolution 2π × 0.06 A^−1^. The phonon frequencies were calculated using the finite displacement approach as implemented in the Phonopy code^46^. The Raman intensities were obtained by computing the derivative of the macroscopic dielectric tensor with respect to the normal mode coordinate^47^.

## Additional Information

**How to cite this article**: Lobanov, S. S. *et al.* Stable magnesium peroxide at high pressure. *Sci. Rep.* **5**, 13582; doi: 10.1038/srep13582 (2015).

## Supplementary Material

Supplementary Information

## Figures and Tables

**Table 1:** Experiments description.

Type	#	Precursor	Culet size, μm	Maximum pressure, GPa	*I4/mcm* MgO_2_	Pressure calibrant
A	1	MgO + O_2_	300/80	96	Yes	MgO
A	2	MgO + O_2_	200	104	Yes	MgO
B	1	MgO + Mg+ MgO_2_	200	70	No	MgO, Au
B	2	MgO + Mg+ MgO_2_	300/100	160	Yes	MgO, Au
