# Supplementary material for: Stable magnesium peroxide at high pressure
Source: Sci Rep. 2015 Sep 1;5:13582. doi: 10.1038/srep13582 (PMC4555032; doi:10.1038/srep13582)
Supplement: Supplementary Information [file srep13582-s1.pdf]

**SUPPLEMENTARY INFORMATION**

# Stable magnesium peroxide at high pressure

Sergey S. Lobanov[1,2,*], Qiang Zhu[3], Nicholas Holtgrewe[1,4], Clemens Prescher[5], Vitali B. Prakapenka[5], Artem Oganov[3,6,7], Alexander F. Goncharov[1,8,9]

[1]Geophysical Laboratory, Carnegie Institution of Washington, Washington, DC 20015, USA

[2]V.S. Sobolev Institute of Geology and Mineralogy SB RAS, Novosibirsk 630090, Russia

[3]Department of Geosciences, Department of Physics and Astronomy, Stony Brook University, Stony Brook, NY 11794, USA

[4]Howard University, 2400 Sixth Street NW, Washington, DC 20059, USA

[5]Center for Advanced Radiation Sources, University of Chicago, Chicago, IL 60632, USA

[6]Moscow Institute of Physics and Technology, 9 Institutskiy lane, Dolgoprudny city, Moscow Region, 141700, Russian Federation

[7]School of Materials Science, Northwestern Polytechnical University, Xi'an,710072, China

[8]Key Laboratory of Materials Physics, Institute of Solid State Physics, CAS, Hefei, 230031, China

[9]University of Science and Technology of China, Hefei, 230026, China

*slobanov@carnegiescience.edu

## Supplementary Tables

**Supplementary Table S1.** Lattice parameters of *I4/mcm* $MgO_2$

| Run # | P, GPa | | | a, Å | σ (a, Å) | c, Å | σ (c, Å) | V, (Å3) | σ (V, $Å^3$) |
|---|---|---|---|---|---|---|---|---|---|
| | MgO | | Au | | | | | | |
| | Ref.[1] | Ref.[2] | Ref.[3] | | | | | | |
| A1 | 93.5 | **95.6** | - | 3.9994 | 0.0009 | 4.7458 | 0.0032 | 75.9104 | 0.0482 |
| A1 | 94.0 | **96** | - | 3.9988 | 0.0010 | 4.7343 | 0.0031 | 75.7028 | 0.0417 |
| A1 | 95.5 | **97.6** | - | 3.9973 | 0.0014 | 4.7271 | 0.0034 | 75.5300 | 0.0501 |
| A1 | 95.7 | **97.8** | - | 3.9961 | 0.0008 | 4.7243 | 0.0038 | 75.4409 | 0.0501 |
| A1 | 97 | **99.2** | - | 3.99161 | 0.0008 | 4.7181 | 0.0031 | 75.1726 | 0.0469 |
| A2 | 101.5 | **104** | - | 3.9787 | 0.0001 | 4.7188 | 0.0004 | 74.6971 | 0.0058 |
| A2 | 102.5 | **105** | - | 3.9803 | 0.0001 | 4.7115 | 0.0004 | 74.6421 | 0.0058 |
| B2 | 130.0 | **134.3** | - | 3.9211 | 0.0007 | 4.6040 | 0.0027 | 70.7882 | 0.0375 |
| B2 | 131.5 | **135.9** | - | 3.92225 | 0.0008 | 4.6026 | 0.0027 | 70.8061 | 0.0376 |
| B2 | 139.0 | **144** | 149,9 | 3.8985 | 0.0007 | 4.5605 | 0.0027 | 69.3124 | 0.0363 |
| B2 | 154.0 | **159.9** | 161.1 | 3.8666 | 0.0013 | 4.5646 | 0.0038 | 68.2455 | 0.0559 |
| B2* | 152.0 | **158** | 156.8 | 3.8862 | 0.0009 | 4.5502 | 0.0028 | 68.7188 | 0.0362 |
| B2* | 150.0 | **155.8** | 152.9 | 3.8909 | 0.0014 | 4.5849 | 0.0033 | 69.4122 | 0.0458 |
| B2* | 146.5 | **152.1** | 151.6 | 3.9060 | 0.0008 | 4.5529 | 0.0035 | 69.4645 | 0.0535 |
| B2* | 116.5 | **119.9** | 116.5 | 3.9451 | 0.0010 | 4.6359 | 0.0030 | 72.1509 | 0.0450 |
| B2* | 115.0 | **118.3** | - | 3.9666 | 0.0009 | 4.6063 | 0.0028 | 72.4751 | 0.0385 |
| B2* | 114.5 | **117.8** | 109 | 3.9619 | 0.0010 | 4.6631 | 0.0031 | 73.1970 | 0.0459 |
| B2* | 112.0 | **115.1** | 111.5 | 3.9617 | 0.0013 | 4.6617 | 0.0039 | 73.1655 | 0.0612 |
| B2* | 110.0 | **113** | - | 3.9800 | 0.0010 | 4.6614 | 0.0031 | 73.8394 | 0.0463 |
| B2* | 108.0 | **110.9** | - | 3.9718 | 0.0013 | 4.6822 | 0.0040 | 73.8614 | 0.0620 |
| B2* | 103.0 | **105.6** | 104.8 | 3.9831 | 0.0014 | 4.7070 | 0.0040 | 74.6762 | 0.0630 |
| B2* | 83.5 | **85** | - | 4.0478 | 0.0012 | 4.8181 | 0.0034 | 78.9424 | 0.0520 |
| B2* | 83.0 | **84.4** | - | 4.0299 | 0.0014 | 4.8199 | 0.0042 | 78.2748 | 0.0676 |
| B2* | 82.5 | **83.9** | - | 4.0517 | 0.0012 | 4.8096 | 0.0034 | 78.9547 | 0.0519 |
| B2* | 74.0 | **75** | 74.2 | 4.0784 | 0.0019 | 4.8687 | 0.0040 | 80.9809 | 0.0585 |

Pressure was gauged using MgO EOS by Speziale et al. [1], Tange et al. [2], and Au EOS is after Fei et al. [3]. Pressure values used to construct *I4/mcm* MgO2 EOS are shown in bold. Decompression run is marked with an asterisk.

**Supplementary Table S2.** Computed lattice parameters of *I4/mcm* $MgO_2$

| P, GPa | a, Å | c, Å |
|---|---|---|
| 70 | 4.0853 | 4.8762 |
| 80 | 4.0572 | 4.8277 |
| 90 | 4.0315 | 4.7836 |
| 100 | 4.0076 | 4.7409 |
| 110 | 3.9855 | 4.7021 |
| 120 | 3.9649 | 4.6662 |
| 130 | 3.9455 | 4.6325 |
| 140 | 3.9272 | 4.6004 |
| 150 | 3.9101 | 4.5697 |

**Supplementary Table S3.** Experimentally and theoretically (DFT) derived parameters of the 300 K third-order Birch-Murnaghan EOS of *I4/mcm* $MgO_2$

| | $V_0$, Å$^3$ | $K_0$, GPa | $K_0$` |
|---|---|---|---|
| Compression | 105.4 | 151.6 | 4 |
| Decompression | 110.1 | 127.1 | 4 |
| DFT | 106.8 | 147 | 4.1 |
| MgO (Ref.[1]) | 74.71 | 160.2 | 3.99 |
| MgO (Ref.[2]) | 74.698 | 160.64 | 4.221 |

## Supplementary Figures

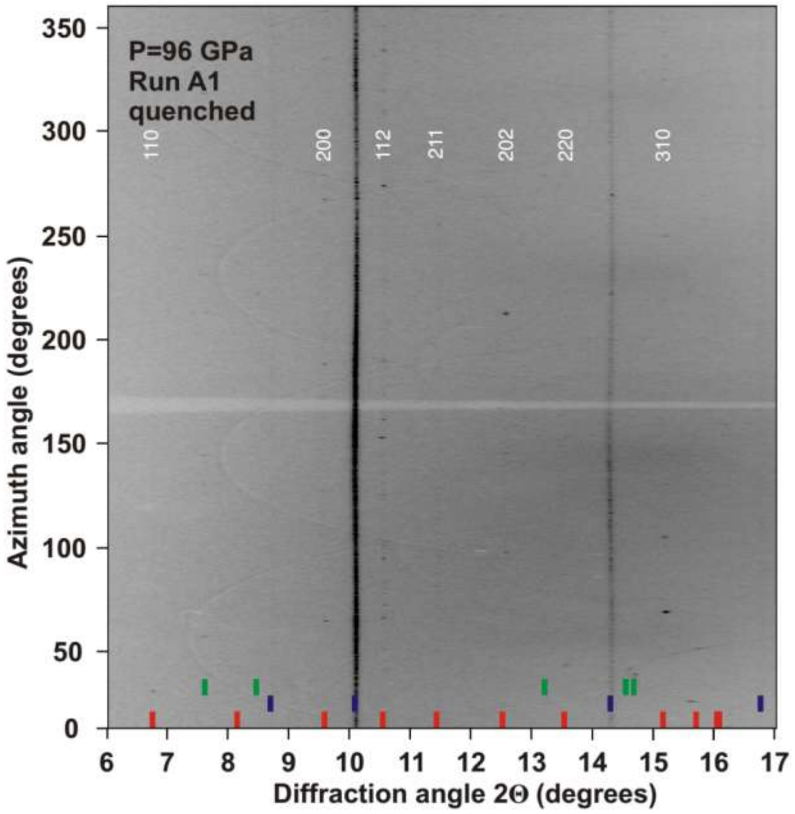


***Supplementary Figure S1.*** *XRD image (cake) of I4/mcm $MgO_2$ synthesized at 96 GPa from the mixture of MgO and $O_2$. Red and violet ticks correspond to the positions of I4/mcm $MgO_2$ and MgO, respectively. Green ticks represent spotty reflections of ζ-$O_2$. White labels are Miller indices of the indexed tetragonal phase. A slight curvature of the vertical lines (originating from MgO is due to a pressure gradient in the sample cavity). The x-ray wavelength is 0.3344 Å.*

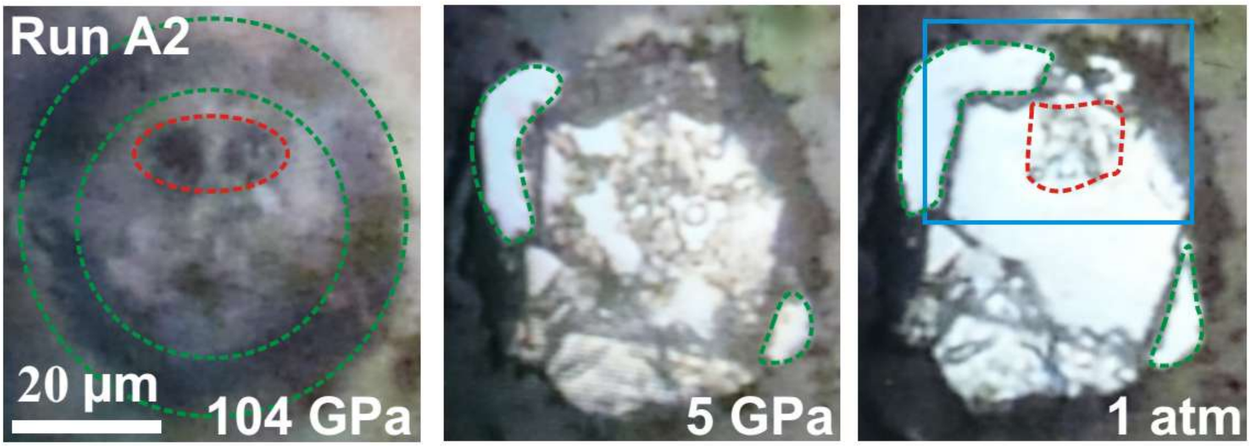


***Supplementary Figure S2.*** *Optical images of the A2 sample in a DAC cavity. Laser-heated area (red dashed line) is shielded from Re gasket by oxygen (green dashed lines). Note the transition from the metallic ζ-$O_2$ at*

*104 GPa (opaque) to transparent at 5 GPa. At 1 atm oxygen was no longer trapped in the DAC. Blue solid line corresponds to the sample area shown in the Figure 5.*

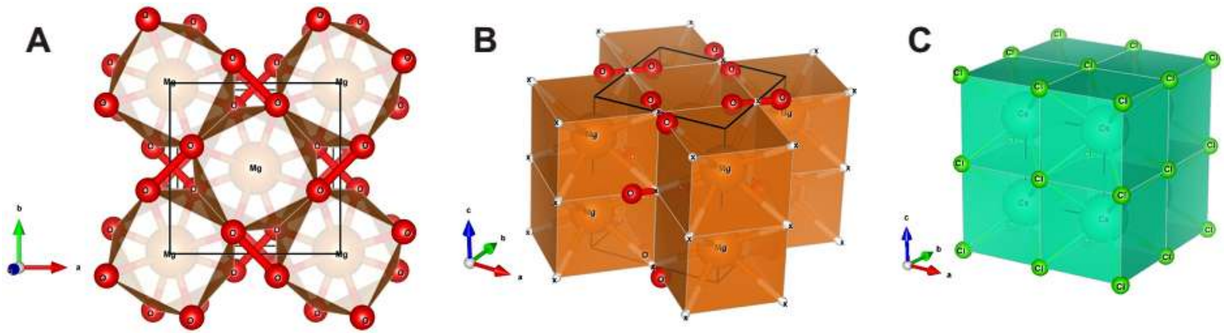


***Supplementary Figure S3.*** *The crystal structure of I4/mcm* $MgO_2$ *(panels **A** and **B**) as compared to CsCl-type (**C**). Lattice positions marked with an x in panel B are centers of the O-O dumbbells. Black solid lines represent unit cells. Crystal structures were visualized by S.S.L. using VESTA v.3 (Ref.[4]).*

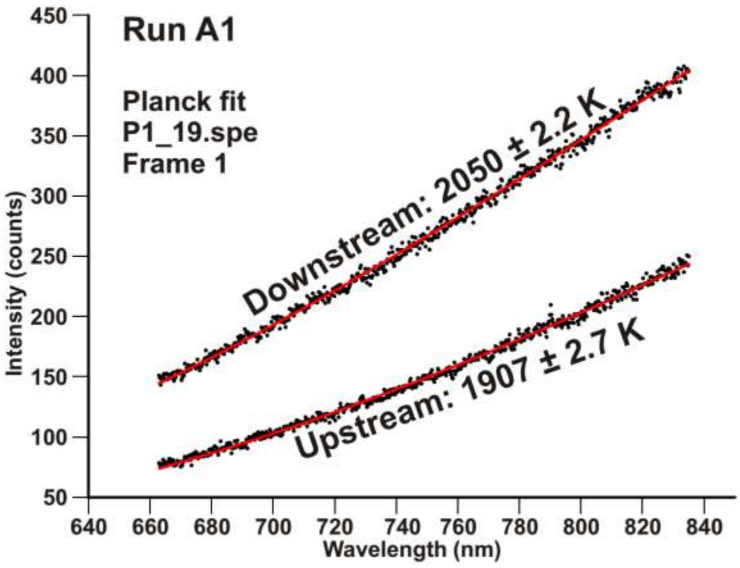


***Supplementary Figure S4.*** *Planck fits (red curves) to the collected thermal radiation (black dots). Only statistical uncertainty related to the fit quality is shown.*

## Supplementary References

1. *Speziale, S., Zha, C. S., Duffy, T. S., Hemley, R. J., Mao, H. K. Quasi-hydrostatic compression of magnesium oxide to 52 GPa: Implications for the pressure-volume-temperature equation of state. Journal of Geophysical Research,* ***106,*** *515-528 (2001).*

2. *Tange, Y., Nishihara, Y., Tsuchiya, T. Unified analyses for P-V-T equation of state of MgO: A solution for pressure-scale problems in high P-T experiments. J Geophys Res-Sol Ea,* ***114,*** *(2009).*

3. *Fei, Y. W., et al. Toward an internally consistent pressure scale. Proc. Natl. Acad. Sci. U.S.A.,* ***104,*** *9182-9186 (2007).*

4. *Momma, K. & Izumi, F. VESTA 3 for three-dimensional visualization of crystal, volumetric and morphology data. J. Appl. Crystallogr.,* ***44,*** *1272-1276 (2011).*
